# Zinc adjunct therapy reduces case fatality in severe childhood pneumonia: a randomized double blind placebo-controlled trial

**DOI:** 10.1186/1741-7015-10-14

**Published:** 2012-02-08

**Authors:** Maheswari G Srinivasan, Grace Ndeezi, Cordelia Katureebe Mboijana, Sarah Kiguli, Gabriel S Bimenya, Victoria Nankabirwa, James K Tumwine

**Affiliations:** 1 Department of Paediatrics and Child Health, School of Medicine, Makerere University, College of Health Sciences, Kampala, Uganda; 2 School of Biomedical Sciences, Makerere University College of Health Sciences, Kampala, Uganda; 3 Department of Epidemiology, Mailman School of Public Health, Columbia University, New York, NY 10032, USA

## Abstract

**Abstract:**

**Background**

Pneumonia is a leading cause of children's deaths in developing countries and hinders achievement of the fourth Millennium Development Goal. This goal aims to reduce the under-five mortality rate, by two thirds, between 1990 and 2015.

Few studies have examined the impact of zinc adjunct therapy on the outcome of childhood pneumonia. We determined the effect of zinc as adjunct therapy on time to normalization of respiratory rate, temperature and oxygen saturation. We also studied the effect of zinc adjunct therapy on case fatality of severe childhood pneumonia (as a secondary outcome) in Mulago Hospital, Uganda.

**Methods**

In this double blind, randomized, placebo-controlled clinical trial, 352 children aged 6 to 59 months, with severe pneumonia were randomized to zinc (20 mg for children ≥12 months, and 10 mg for those < 12 months) or a placebo once daily for seven days, in addition to standard antibiotics for severe pneumonia. Children were assessed every six hours. Oxygen saturation was normal if it was above 92% (breathing room air) for more than 15 minutes. The respiratory rate was normal if it was consistently (more than 24 hours) below 50 breaths per minute in infants and 40 breaths per minute in children above 12 months of age. Temperature was normal if consistently below 37.5°C. The difference in case fatality was expressed by the risk ratio between the two groups.

**Results**

Time to normalization of the respiratory rate, temperature and oxygen saturation was not significantly different between the two arms.

Case fatality was 7/176 (4.0%) in the zinc group and 21/176 (11.9%) in the placebo group: Relative Risk 0.33 (95% CI 0.15 to 0.76). Relative Risk Reduction was 0.67 (95% CI 0.24 to 0.85), while the number needed to treat was 13. Among HIV infected children, case fatality was higher in the placebo (7/27) than in the zinc (0/28) group; RR 0.1 (95% CI 0.0, 1.0).

Among 127 HIV uninfected children receiving the placebo, case fatality was 7/127 (5.5%); versus 5/129 (3.9%) among HIV uninfected group receiving zinc: RR 0.7 (95% CI 0.2, 2.2). The excess risk of death attributable to the placebo arm (Absolute Risk Reduction or ARR) was 8/100 (95% CI: 2/100, 14/100) children. This excess risk was substantially greater among HIV positive children than in HIV negative children (ARR: 26 (95% CI: 9, 42) per 100 versus 2 (95% CI: -4, 7) per 100); *P*-value for homogeneity of risk differences = 0.006.

**Conclusion**

Zinc adjunct therapy for severe pneumonia had no significant effect on time to normalization of the respiratory rate, temperature and oxygen saturation. However, zinc supplementation in these children significantly decreased case fatality.

The difference in case fatality attributable to the protective effect of zinc therapy was greater among HIV infected than HIV uninfected children. Given these results, zinc could be considered for use as adjunct therapy for severe pneumonia, especially among Highly Active Antiretroviral Therapy

naïve HIV infected children in our environment.

**Clinical trials registration number:**

clinicaltrials.gov NCT00373100

## Background

Acute respiratory tract infections are the most common cause of morbidity and deaths in children less than five years. They account for 10 to 30% of all childhood deaths and are thus a hindrance to attaining the fourth Millennium Development Goal [1]. The burden of acute lower respiratory tract infections is 2 to 10 times more common in developing than in developed countries [2].

Zinc deficiency is a global problem affecting populations of low socioeconomic status in both developing and developed countries [3]. Populations in South-East Asia and sub-Saharan Africa are at greatest risk because zinc intakes are inadequate for about one-third of the population [4]. In addition, zinc is the most deficient nutrient in complementary foods fed to infants during weaning [5]. The prevalence of zinc deficiency in Uganda ranges from 20 to 69% in children and 21 to 29% in adults [6,7].

One randomized controlled study from Bangladesh showed that zinc adjunct therapy accelerated recovery of children with severe pneumonia [8], but other studies have shown no effect [9,10]. Hitherto, no studies have assessed the impact of zinc adjunct therapy on case fatality of children with pneumonia.

The main objectives of our study were to establish time taken for normalization of respiratory rate, time taken for normalization of temperature, and time taken for oxygen saturation to normalize. The secondary objective was to determine the effect of zinc adjunct therapy on case fatality of severe childhood pneumonia in children aged 6 to 59 months at Mulago Hospital in Uganda. The major and very interesting finding from this study (that is, the effect of zinc adjunct therapy on severe pneumonia case fatality) comes from the secondary objective and not from any of the primary ones.

## Methods

### Study design and setting

This was a randomized double blind placebo-controlled clinical trial. The study was conducted in one pediatric ward at Mulago, Uganda's teaching and national referral hospital. It receives patients from the capital city, Kampala, and referrals from the rest of the country.

### Outcome measures

The primary outcome measures were the time taken for normalization of respiratory rate, time taken for normalization of temperature and time taken for oxygen saturation to normalize (92% or more), while breathing room air. The oxygen saturation cut off was set at 92% according to a study in the same hospital by Tumwesigye [11]. The secondary outcome measure *was *the proportion of children who died in each arm of the study.

Adverse effects due to zinc adjunct therapy, such as diarrhea and vomiting, were recorded. All children were observed for adverse events for one hour after zinc administration. The caretakers were subsequently asked (daily) about adverse events, such as vomiting.

### Definitions

Severe pneumonia was defined as the presence of cough or difficult breathing with fast breathing (tachypnoea) and chest in-drawing. Tachypnoea was defined as a respiratory rate of 50 or more breaths per minute in a child 6 to 12 months of age. For those aged 12 or more months it was defined as 40 or more breaths per minute. In any case, the child had to be calm. Normalization of oxygen saturation was regarded as oxygen saturation above 92% while breathing room air for more than 15 minutes, and maintaining above 92% on subsequent readings. Children, who were not hypoxic on enrolment, were assigned 'zero' as the time to normalization of oxygen saturation.

The respiratory rate was regarded as normal if it was consistently below 50 breaths per minute in infants and 40 breaths per minute in children above 12 months of age for more than 24 hours. Temperature was recorded as normal if below 37.5°C and maintained below this level for more than 24 hours. Two investigators agreed on the presence of lower chest retractions or chest in-drawing. If they disagreed, a third investigator was called to observe and decide whether chest in-drawing was present. When two of the three agreed, then the decision was that chest in-drawing was present.

### Sample size

For time to normalization of the respiratory rate, a sample size of 313 was calculated. We assumed 90% power, a two-sided 0.05 type I error and a hazard ratio of 1.49. We assumed a median time to normalization of respiratory rate of 73 hours (based on an Indian study) [9]. Allowing for 10% attrition, the minimum total sample size was 344.

For time to normalization of the temperature, a sample size of 312 was calculated. We assumed 90% power, a two-sided 0.05 type I error and hazard ratio of 1.49; and a median time to normalization of the temperature of 72.3 hours (based on the same Indian study) [9]. Allowing for 10% attrition the minimum sample size was 343. The sample size for time to normalization of oxygen saturation was similar to that of time to normalization of the temperature. For case fatality as a secondary outcome, the minimum sample size of 378 (189 in each group) was based on 20% case fatality in children admitted with severe pneumonia to Mulago Hospital [12]. We assumed that zinc supplementation would reduce the case fatality from 20% to 9.3% with 80% power and a type 1 error of 0.05. Allowing for 10% attrition, the minimum total sample size would be 416 (208 in each group).

### Participants and recruitment

We enrolled children, aged 6 to 59 months, who were admitted to the Mulago Hospital pediatric emergency ward with severe pneumonia, and whose parents or caretakers gave informed written consent. The children were followed up for one week. The definition of severe pneumonia was based on WHO criteria [13].

Children with known heart conditions, obstructive air way disease and those on medication with zinc supplements were excluded. Children with a current episode of wheeze were also excluded, irrespective of previous wheezing attacks. The exclusions were based on caretaker history, physical examination findings and previous medical records. Heart disease was defined as the presence of tachypnoea associated with a grade 3 or 4 heart murmur or medical records confirming heart disease. Obstructive airway disease was defined as recurrent wheezing attacks associated with tachypnoea or chest in-drawing confirmed on two or more previous examinations by medical records. Current treatment with zinc was confirmed by reviewing medical records.

For all enrolled children, two of us (MGS and CKM) took a detailed history and clinical examination.

A pulse-oximeter (model Ohmeda Biox 3700 Ohmeda (GE Healthcare), Pollards Wood Chalfont St. Giles, Bucks HP8 4SP United Kingdom.) with an in-built automatic calibration was used to measure oxygen saturation. Blood smears for malarial parasites and HIV serostatus of the children were also assessed. Additional consent was sought for HIV testing. Only 311/352 or (88.4%) consented to HIV testing.

### Randomization, blinding and treatment allocation

Eligible children were enrolled in serial order and randomized to receive either oral zinc or placebo.

The zinc and placebo tablets were packaged and labeled with study identification numbers by a pharmacist not directly involved in the care of the patients. Macleod Pharmaceutical Ltd, Mumbai, India, manufactured the zinc and placebo tablets. Both zinc and placebo tablets were similar in color, shape and size. The investigators, study nurses and parents/caretakers were masked to the treatment and only the pharmacist had access to the study code. On receiving an eligible child, the study physicians informed one of the investigators (pediatrician) who in turn informed the pharmacist who had access to the randomization code. The pharmacist then prepared the dose of zinc/placebo according to the age of the participant. He packaged the zinc or placebo in a sealed opaque envelope, which he labeled with the study identification number.

The envelopes were then delivered to one of the investigators' offices from where the nurse took them to the ward. The nurse gave treatment according to the envelopes. Zinc/placebo administration was directly supervised by the nurse within 30 minutes after giving antibiotics.

### Intervention

Children less than 12 months received 10 mg of zinc (as zinc gluconate) or the placebo while those aged 12 months or more received 20 mg of zinc or placebo once daily for seven days as we anticipated children with pneumonia to have recovered [14] by Day 7. Dosing was closely related to the recommended dietary allowance (RDA) of zinc [15]. The water soluble tablets were crushed into powder and placed into the mouth of those children who could not take them directly. If vomiting occurred within half an hour, the dose was repeated.

### Laboratory and radiological investigations

Blood for full blood count and HIV serology was taken by venipuncture from the cubital fossa or dorsum of the hand in 5 ml EDTA vacutainer tubes (Becton Dickinson, Franklin Lakes, NJ, USA). HIV sero-status of study patients below 18 months of age was confirmed using DNA PCR. Blood for serum zinc was collected using trace element-free vacutainers (Becton Dickinson) and storage tubes.

Just before the zinc assays were carried out, we diluted the serum samples five-fold using de-ionized water according to the manufacturer of the test kit [16]. Colorimetric zinc determination was done using a Quantichrom™ zinc assay kit (BioAssay Systems · 3191 Corporate Place, Hayward, CA 94545, USA). In this assay, serum zinc reacts with a chromogen to give a colored complex whose color intensity is directly proportional to the zinc concentration read at 425 nm. Internal control was used, using pooled serum and every batch produced a value of 45.2 ± 4.5 μg/dL. Unfortunately, no external controls were available.

We transferred 50 μL of water, zinc standard (10 μM), sample and sample Blank (50 μL sample + 2 μL EDTA) into wells of a clear bottom 96-well plate according to the manufacturer's manual [16]. We then added 200 μL working reagent, tapping the plate gently in order to mix. This was incubated for 30 minutes at room temperature and the optical density was read at 420 to 426 nm (peak absorbance at 425 nm).

The concentration of zinc was then calculated using the following equation:

ODSAMPLE-ODBLANK,×10×n(micromoles)/ODSTANDARD-ODWATER

OD is the optical density at 425 nm. Where n is the dilution factor (n = 5 for serum or plasma samples). The conversion was ''1 μM zinc equals 6.5 μg/dL.''

### Treatment of participants

Children received either intravenous chloramphenicol[Ciron Drugs &Pharceuticals Ltd, MIDC, Tarapur Boosar Thane India] 25 mg/kg every six hours or ceftriaxone [Hoffmann Roche Ltd Basel, Switzerland] 75 mg/kg once daily for seven days. Second line antibiotics: gentamicin (2.5 mg/kg every 12 h) and cloxacilin [Flamingo Pharmaceuticals Ltd, Indus, India]. (50 mg/kg every 6 h) were only given if there was no response after two days of treatment. On enrolment, 50/352 (14.2%) children were unable to feed orally and fed by naso-gastric tube on milk, porridge and locally available soups. Children with a peripheral oxygen saturation of less than 92% were given oxygen by nasal prongs or catheter.

For patients on oxygen, we disconnected the oxygen supply for five minutes before the oxygen saturation was measured. We counted respirations using a timer for 60 seconds when the chest and abdomen were exposed according to WHO Integrated Management of Childhood Illness (IMCI) guidelines [13]. The count was repeated and the average was taken as the true value if the difference between the two counts was not more than five breaths per minute. If the difference was more than five breaths per minute, a third count was done and the average of the closest two respiratory rates was taken as the true value.

The study team was comprised of three doctors and two nurses (clinic team) and two laboratory personnel. The clinic team had prior experience in assessing and managing children with severe pneumonia. They were trained on the study procedures and assessment of clinical signs before commencement of the study to ensure reproducibility and consistency.

A three-member independent team was comprised of a pediatrician, an epidemiologist and a pharmacist, who regularly and closely monitored the data. Interim analysis was done when we had accrued half the sample size. All children received vitamin A as part of treatment for pneumonia as recommended by WHO [13]. Fluid therapy was given according to hydration status.

### Follow-up

All children were followed up until discharge, death or a maximum of seven days, whichever came first. Patients were reviewed every six hours for the first 48 hours and then every 12 hours. The clinical review included the respiratory rate, presence of chest in-drawing, oxygen saturation, auscultation findings, fever, cyanosis and mental state. All children were monitored for vomiting and diarrhea using information from the caretaker or by observation. Criteria for discharge included: resolution of fever, persistently normal respiratory rate, and oxygen saturation for more than 48 hours and disappearance of chest in-drawing.

### Data management and analysis

• All the data were double-entered into the EPI-INFO 6.04 computer software package (Center for Disease Control and Prevention, Atlanta, Georgia, USAand analyzed using SPSS(SPSS Inc., Chicago Ill.)

= and Stata version 9.2 (StataCorp LP, College Station, TX, USA). Analysis was by intention to treat. The Student's *t*-test was used for comparing normally distributed continuous data, and the Mann Whitney test for skewed continuous data. The Chi square or Fisher's exact test (where appropriate) was used to assess differences in categorical data between the zinc and placebo arms. Each child's observation started on enrolment and ended on Day 7 or earlier, at the time of death for those who died. Participants who died before normalization of the primary study outcome, such as the respiratory rate, contributed person time of observation. Those who died after the normalization of the primary study outcome, such as the respiratory rate, were not censored for that outcome.

We used risk ratios (RR) to measure the association between case fatality and the intervention. We used multivariable generalized linear model (GLM) regression analysis with a log link to estimate the adjusted RR of the intervention on case fatality.

Initially, we estimated the effect of wasting (WHZ), percentage oxygen saturation, HIV status, age, relationship with the caretaker and blood culture results on case fatality in crude analyses. However, only variables that were associated with case fatality with a *P*-value < 0.25 or whose addition resulted in a 10% difference in the parameter estimates, were included in the final multivariable model.

A cross product term (for HIV status and zinc) was included in one of the multivariable models to test for interaction on the multiplicative scale. We examined interaction on the additive scale by estimating homogeneity of stratum specific risk differences. Cox proportional hazards models were used to compare time to normalization of respiratory rate, temperature and oxygen saturation in the two arms.

### Ethical issues

Permission to conduct the study was obtained from Makerere University Faculty of Medicine and Mulago Hospital Ethics committees.

## Results

**Figure 1:**
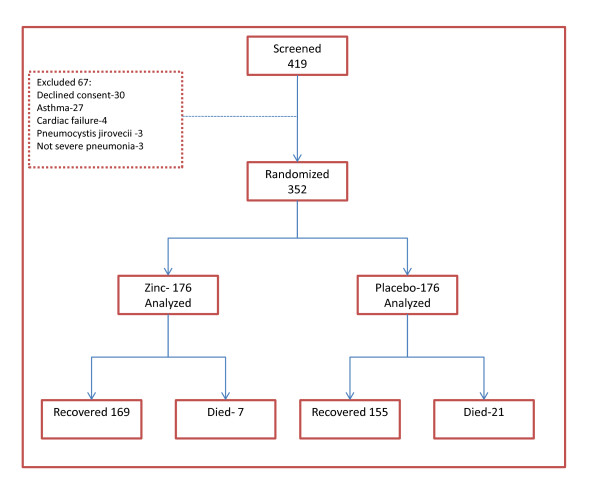
**Study profile**.

From September 2006 to March 2007, 475 children with severe pneumonia were screened for this study. Of these, 352 were recruited into the study: 176 were randomized to zinc, while 176 received the placebo (Figure 1).

### Description of study subjects

Of the 352 participants, 198 (56.2%) were male. Age ranged from 6 to 59 months with a mean of 17.9 (SD 12.2) in the zinc group and 18.1 (SD 11.7) for the placebo group. The rest of the baseline characteristics are shown in Table 1. Sixty-two (18.1%) children had low oxygen saturation below 92%. The mean respiratory rate was 64.7 (SD 15.7), with 65.6 (SD 15.1) in the zinc group and 63.9 (SD 16.3) in the placebo group.

**Table 1:** Baseline characteristics of children admitted with severe pneumonia, Mulago Hospital

Variable	Zinc group N = 176 N (%)	Placebo group N = 176 N (%)
Sex: Female	78 (44.3)	76 (43.2)
Age in months 6 to 12	76 (43.2)	68 (38.6)
13 to 59	100 (56.8)	108 (61.4)
Mean age (SD)	17.9 (12.2)	18.1 (11.8)
Care taker: Mother	152 (87.4)	163 (94.2)
Other	22 (12.6)	10 (31.3)
Distance: < 5 km	65 (39.4)	70 (41.7)
≥5 km	100 (60.6)	98 (58.3)
Breastfeeding at six months Yes	40 (80.0)	39 (73.6)
No	10 (20.0)	14 (26.4)
Measles vaccination: Yes	103 (83.1)	107 (80.5)
No	21 (16.9)	26 (19.6)
Inability to feed (%)	25 (14.3)	25 (14.2)
Duration of cough in days (IQR)	7.0 (4.0 to 14.0)	7.0 (3.0 to 14.0)
Oxygen saturation (IQR)	95.0 (91.0 to 97.0)	94.0 (90.0 to 98.0)
Oxygen saturation at enrolment < 92%	48 (27.7)	54 (31.4)
≥92%	125 (72.3)	118 (68.6)
Crepitations: Yes	140 (80.0)	147 (84.0)
No	35 (20.0)	28 (16.0)
WHZ <-2SD	30 (17.1)	40 (22.9)
≥-2SD	146 (83.0)	135 (77.1)
HAZ <-2SD	46 (26.1)	44 (25.1)
≥-2SD	130 (73.9)	131 (74.9)
HIV Positive	28 (17.8)	27 (17.5)
Negative	129 (82.2)	127 (82.5)
Malaria (n = 343): Positive	18 (10.5)	19 (11.0)
Negative	153 (89.5)	153 (89.0)
Blood culture: positive	19(20.2)	26(28.9)
negative	75(79.8)	64(71.1)
Zinc in μmol/L (IQR)	4.4 (1.3 to 8.0)	4.8 (2.3 to 10.4)

HAZ, height for age z score; IQR, inter-quartile range; WAZ, weight for age z score

### Laboratory results

The laboratory results included blood slide examination for malarial parasites, HIV serology and chest radiographic findings. A total of 311 children were screened for HIV: 55 (17.7%) were positive; 28/157 (17.8%) in the zinc group and 7/154 (17.5%) in the placebo group.

Overall, 45/184 (24.5%) of the blood cultures were positive for bacteria. The bacteria identified included *Streptococcus pneumoniae *(14), *Staphylococcus aureus *(16), *Salmonella species *(7), *Listeria monocytogenes *(3), *Haemophilus influenzae *(2) and others (3): that is, *Klebsiella pneumoniae*, ***Pseudomonas aeruginosa ***and *E.coli*. The rest of the laboratory findings are shown in Table 1.

### Outcome measures

#### Time to normalization of respiratory rate

The median time for normalization of respiratory rate was 96 hours in the zinc group and 86 hours in the placebo group. The difference was not statistically significant (Table 2).

**Table 2:** Time to normalization of parameters of disease severity, among children with severe pneumonia

Outcome	Zinc	Placebo		
	**Median (95% CI)**	**Median (95% CI)**	**Hazard ratio (95% CI)**	***P-*value**
**Time to normalization of respiratory rate (hours)**	96.0 (83.0, 109.0)	86.0 (75.4, 96.6)	0.88 (0.69, 1.13)	**0.306**
**Time to normalization of temperature (hours)**	18.0 (15.1, 20.9)	18.0 (16.0, 20.0)	1.016 (0.79, 1.30)	**0.897**
**Time to normalization of oxygen saturation (hours)**	**24. 0 (20.6, 27.4)**	**18.0 (10.6, 25.4)**	**1.04 (0.74, 1.46)**	**0.823**

#### Time to normalization of temperature

Median time to normalization of the temperature was 18.0 hours in both the zinc and placebo arms (Table 2).

#### Time to normalization of oxygen saturation

The median time to normalization of oxygen saturation was 24 hours in the zinc group and 18 hours in the placebo arm (Table 2).

### Duration of hospitalization

Duration of hospitalization was 2.57 days (95% CI 1.72, 3.43) among those who died, and 6.91 (95% CI 6.85, 6.97) for those who survived.

There was no interaction between zinc and HIV status on the time to normalization of respiratory rate, temperature and oxygen saturation.

### Case fatality

Overall case fatality was 28/352 (8.0%): 7/176 (4.0%) in the zinc and 21/176(11.9%) in the placebo group. Case fatality was lower in the zinc group: Relative Risk (RR) 0.33 (95% CI of 0.15 to 0.76). When we stratified case fatality by HIV status, the effect of zinc supplementation appeared stronger in the HIV infected subgroup.

Among 27 HIV-infected, Highly Active Antiretroviral Therapy naive children receiving placebo, case fatality was 7/27 (25.9%); versus 0/28 (0%) among HIV infected children receiving zinc: RR 0.1 (95% CI 0.0, 1.0). Among 123 HIV uninfected children receiving placebo, case fatality was 7/127 (5.5%); versus 5/129 (3.9%) among HIV uninfected children receiving zinc: RR 0.7 (95% CI 0.2, 2.2).

The Relative Risk Reduction (RRR) was 0.67 (0.24 to 0.85) while the number needed to treat (NNT) was 13. The excess risk of death attributable to the placebo arm (Absolute Risk Reduction or ARR) was 8/100 (95% CI: 2/100, 14/100) children. This excess risk was substantially greater among HIV positive children than in HIV negative children (ARR: 26 (95% CI: 9, 42) per 100 versus 2 (95% CI: -4, 7) per 100); *P*-value for homogeneity of risk differences = 0.006.

### Other factors predicting case fatality

HIV-positive children had a higher risk of death compared to HIV-negative children: RR 2.6 (95% CI 1.0, 6.7). This increased risk was mostly attributable to the subgroup of HIV-positive children that received the placebo: RR 4.7 (95% CI 1.8, 12.3) as compared to children that received the zinc adjunct therapy: RR 0.9 (95% CI 0.1, 7.6).

Children who were hypoxic or malnourished were more likely to die, compared to HIV-negative or healthier children (Table 3). Girls were two times more likely to die compared to boys (RR 2.3, 95% CI: 1.1, 4.9).

**Table 3:** Predictors of case fatality among children with severe pneumonia, Mulago Hospital, Uganda^a ^

Variable	RR (Unadjusted) 95% CI	RR (adjusted)^b ^95% CI	RR (adjusted)^c ^95% CI
Zinc adjunct therapy	0.3 (0.1,0.8)	0.3 (0.1, 0.97)	0.2 (0.0, 1.1)
WHZ			
<-2SD	0.29 (0.1, 0.6)	0.3 (0.1, 0.8)	0.3 (0.1, 0.7)
Oxygen saturation			
< 92%	2.4 (1.1, 5.1)	1.6 (0.6, 3.9)	1.4 (0.6, 3.3)
HIV status			
Positive	2.7 (1.1, 6.6)	2.6 (1.0, 6.7)	4.3 (1.4, 12.7)
Age			
6 to 12	1.0 (0.5, 2.0)		
13 to 24	0.1 (0.0, 1.1)		
Caretaker relationship to child			
Other	0.8 (0.2, 3.2)		
Blood culture positive	1.8 (0.6, 6.0)		
Zinc adjunct therapy* HIV status**^d^**			5.5 (0.5, 58.3)

^a ^CI, confidence interval

^b ^Only variables with a *P*-value less than 0.25 in the unadjusted analysis were included in this model.

^c ^Model includes variables with a *P*-value less than 0.25 and the interaction term for HIV status and Zinc

^d ^Cross-product (interaction) term for HIV status and zinc

We found that 3/26 (11.5%) of the patients with positive blood cultures, and receiving placebo died, while none of the blood culture positive patients receiving zinc died. Case fatality among the blood culture negative patients receiving placebo was 12/64 (18.8%) compared to 5/75 (6.7%) of the blood culture negative patients receiving zinc.

### Adverse drug events

Two children developed vomiting immediately after receiving the first dose of the intervention: one in the zinc group and one in the placebo group. Subsequent doses were tolerated.

## Discussion

We carried out a study to determine the effect of zinc as adjunct therapy on clinical recovery, case fatality and adverse events in children with severe pneumonia in Uganda's national referral hospital. There are two key findings in this study: overall, zinc supplementation in these children significantly decreased case fatality, but did not reduce the time to normalization of the parameters for disease severity.

The children who received the placebo were three times more likely to die as compared to those who received zinc. The number needed to treat was 13. This means that we need to treat 13 children with zinc as adjunct therapy to avert one death. Since the cost of zinc for one child was 630 Ugandan shillings (0.3 USD), we needed 8,190 Ugandan shillings (or 4 USD) to prevent one death. On sub-group analysis the difference in case fatality attributable to the protective effect of zinc therapy was greater among HIV-infected children than among HIV uninfected children. This is not surprising since zinc is known to improve immune response. For example, zinc supplementation might increase phagocytosis [17] and zinc deficiency predisposes to apoptosis of T lymphocytes in HIV-infected patients [18]. In fact, it has now been established that zinc deficiency compromises immunity through a number of mechanisms, such as T cell dysfunction and dysregulation of intracellular killing [19].

We found that 11.5% of the patients with positive blood cultures and receiving the placebo died, while none of the blood culture positive patients receiving zinc died. Case fatality among the blood culture-negative patients receiving the placebo was 18.8% compared to 6.7% of the blood culture-negative patients receiving zinc. Despite the small numbers, zinc reduced case fatality regardless of blood culture results. We did not find a similar study with which to compare our results. In a related study, Cole and Bose [20] used a CRP of > 40 mg/L as a proxy for bacterial pneumonia. They found that zinc therapy for children with severe pneumonia was associated with prolonged hospitalization. They, however, did not study the effect of zinc on case fatality.

Girls were two times more likely to die than boys. The reason for this is not clear. One study from India found pneumonia case fatality was about '50% higher in girls than in boys' [21]. Indeed, excess case fatality rates among girls has been reported by other workers from India who speculated that female children are less likely to be taken for vaccination and medical treatment [22].

In our study, the serum zinc concentrations were low. They are lower than those previously reported in Ugandan children [6,7]. The very low zinc levels could be explained by pre-existing zinc deficiency, redistribution of zinc to the liver in response to pro-inflammatory cytokines, and low albumin concentrations [10]. Children with zinc deficiency have increased susceptibility to bacterial disease and are more likely to die [23]. Animal studies have arrived at similar conclusions [24].

Studies on the effect of zinc supplementation on pneumonia have not reported the prevalence of HIV in the patients studied [10,20]. None the less, the prevalence of HIV in children in our study was high. In a way our HIV-infected children with severe pneumonia suffered from a double disadvantage: HIV related immunodeficiency and that induced by zinc deficiency.

It appears that initial supplementation with zinc modulates this double disadvantage and significantly reduces case fatality. However, the 'modulation' does not appear sufficient to shorten recovery from the pneumonia itself in these HAART naïve, HIV infected children. It is also possible that zinc increases production of pro-inflammatory cytokines which would be expected to exacerbate the situation [25,26]. However, this may not be sufficient to cancel out the benefit of the zinc on case fatality.

In our study, there was no significant difference in the measures of clinical improvement between children receiving the placebo and those receiving zinc, implying that the zinc might have no appreciable role in shortening the duration of severe pneumonia even though it significantly reduces case fatality. What is clear, though, is that we have un-earthed a very interesting, yet contradictory phenomenon, that seems to be related to HIV infection and severe zinc deficiency. It calls for further studies on the interaction among HIV, zinc and severe pneumonia.

Hitherto, zinc had only been conclusively linked to reduction of pneumonia incidence and case fatality when used for long term supplementation and not as adjunct therapy [27]. In a study by Brooks *et al*. in Bangladesh, weekly zinc supplementation for 12 months reduced the incidence of pneumonia. Of the 14 deaths in the Bangladeshi placebo group, 10 were pneumonia-related while there was no pneumonia-related death in the zinc group [27].

Conditions associated with decreased immunity, such as malnutrition, partial immunization and HIV infection, were more likely to be associated with case fatality. For populations where children are deficient in zinc, severe prolonged infections deplete the zinc even further and decrease resistance to infection [28].

### Time to normalization of the parameters for disease severity

The second finding in this study is that zinc supplementation as adjunct therapy for pneumonia did not reduce the time to normalization of the parameters for disease severity. Indeed, the median time for normalization of the respiratory rate, oxygen saturation and temperature in the zinc and placebo groups were not significantly different. This is consistent with results of two Indian and one Australian studies [9,29,30].

However, a Bangladesh study demonstrated a significant difference in time to normalization of respiratory rate (40 to 48 hours in the zinc and placebo groups) [8]. Indeed, in a recent study from Nepal, it was shown that zinc adjunct therapy did not accelerate recovery from non-severe pneumonia [10] and called for larger studies, especially of severe pneumonia. So regarding the effect of zinc on normalization of parameters of disease severity in severe pneumonia, the jury is still out.

### Limitations

There are a few limitations to our study. The median age at death for children with pneumonia in Uganda is 16 months with an interquartile range of 8 to 23 months according to a study by Kallander [14]. Therefore, we enrolled children aged 6 to 59 months into the study, which encompasses this age group. However, one would expect children below the age of six months to have the highest risk of case fatality from pneumonia. So excluding them might have affected our results.

We attempted to get blood culture on most of our patients but did this on only 184/352 (52.3%) of the children. Of these, 45/184 (24.5%) were culture positive. Secondly, we did not measure serum C-reactive protein (CRP), an acute phase protein. We followed up patients for only seven days, yet it is possible that a sizeable proportion had pneumonia for a longer period. In the sample size calculation for case fatality, we arbitrarily chose an HR of 1.49. As it turns out, this was over ambitious and represents an over-optimistic effect.

It might have been unrealistic to expect a reduction of the case fatality from 20% in the placebo group to 9.3% in the zinc group. In the calculation of the sample size, we assumed a case fatality of 20% in the placebo group. This was based on results of previous studies by our team [12] where we demonstrated a case fatality of 20% in children with severe pneumonia. Another one of our studies showed a case fatality of 15.5% among children with severe pneumonia in the same hospital [31].

Of course, these are high case fatalities but were not un-expected, especially since the children were ill with malnutrition and/or HIV.

In our study, 20/351 (4.5%) children died before achieving normalization of the respiratory rate, temperature and oxygen saturation (the numbers and proportions are much smaller when each of these outcomes is considered independently) and were thus lost to follow-up. This loss may have caused bias but, because the loss was minimal, it is unlikely to have been large enough to alter the results significantly. Indeed, these high case fatalities reflect the importance of pneumonia as a cause of child deaths in the community.

In a recent study, Kallander and others in the Iganga Demographic Study Site in eastern Uganda found that pneumonia accounted for 27% of child deaths [14]. We are heartened that the overall case fatality in the current study was only 28/352 (8.0%), dropping to 4.0% in the zinc supplemented group.

Unlike a similar study, which followed up patients for two weeks [10], we followed up patients for a maximum of seven days based on our experience of severe pneumonia patients most of whom spend less than seven days [2,31] in the hospital. The relatively short time of follow-up might have reduced our power to detect a difference in the duration of hospitalization.

## Conclusion

Zinc adjunct therapy for severe pneumonia had no significant effect on time to normalization of the respiratory rate, temperature and oxygen saturation. However, zinc supplementation in these children significantly decreased case fatality. The difference in case fatality attributable to the protective effect of zinc therapy was greater among HIV-infected than among HIV-uninfected children. Given these results, zinc could be considered for use as adjunct therapy for severe pneumonia, especially among HAART naïve, HIV-infected children in our environment.

## Abbreviations

ARR: Absolute Risk Reduction; CI: confidence interval; EDTA: Ethylenediaminetetraacetic acid; HIV: Human immunodeficiency virus; WHO: World Health Organization

## Competing interests

The authors declare that they have no competing interests.

## Authors' contributions

MSG, JKT and GN conceptualized the study. CKM and MSG collected the data and followed up patients. SK, JKT and GN supervised the study. GB supervised the laboratory component. VN, MSG, JKT and GN analyzed and interpreted the data. MSG, JKT and GN wrote the manuscript. All authors have read, revised and approved the manuscript.

## Pre-publication history

The pre-publication history for this paper can be accessed here:

http://www.biomedcentral.com/1741-7015/10/14/prepub
